# Diagnostic Role of Calretinin in Suspicious Cases of Hirschsprung’s Disease

**DOI:** 10.7759/cureus.13373

**Published:** 2021-02-16

**Authors:** Sanjeev K Singh, Umesh K Gupta, Roopak Aggarwal, Rafey A Rahman, Nand K Gupta, Vandana Verma

**Affiliations:** 1 Pathology, Uttar Pradesh University of Medical Sciences, Saifai, Etawah, IND; 2 Pediatric Surgery, Uttar Pradesh University of Medical Sciences, Saifai, Etawah, IND; 3 Anatomy, Uttar Pradesh University of Medical Sciences, Saifai, Etawah, IND; 4 Obstetrics and Gynecology, Uttar Pradesh University of Medical Sciences, Saifai, Etawah, IND

**Keywords:** immunohistochemistry, ganglion cells, congenital aganglionic megacolon, failure to thrive, constipation, functional intestinal obstruction, abdominal distension, aganglionosis

## Abstract

Background

Hirschsprung’s disease (HD) is a developmental disorder of the intrinsic component of the enteric nervous system. It is characterized by the absence of ganglion cells in the myenteric and submucosal plexus. Histopathological diagnosis becomes difficult many times due to submucosal ganglionic cells are not easily identifiable.

Aims and objective

The aim of this study was to examine the clinical and histopathological features of HD and to establish the utility of calretinin staining to diagnose the case of suspicious HD.

Materials and methods

After taking necessary informed consent, we studied 41 cases in which clinical suspicion of HD had been made, in a study duration of three years (July 2017-June 2020). Open biopsies were taken from spastic segment, transition zone and dilated segment. Histopathological diagnosis had been made in three categories: HD, no Hirschsprung's disease (NHD) and suspicion of HD. Post histopathological diagnosis calretinin immunohistochemistry (IHC) was applied to all cases and interpretations were noted.

Results

On the basis of histopathological findings, 25 cases were diagnosed as HD, nine cases were marked for suspicion for HD and seven cases as NHD. After evaluating calretinin IHC on the suspicious case, total of 30 cases were confirmed as HD while the remaining 11 cases were confirmed as NHD. Twenty-four patients of HD were males while the remaining six cases were females. The age of patients ranged from four days to 10 years. Median age six days while 22 patients were in the neonatal period.

Conclusion

Calretinin immunostaining is a useful modality in diagnosing suspicious cases of HD. Its results are easy to interpret by less experienced pathologist with accuracy.

## Introduction

Hirschsprung’s disease (HD) is also known as congenital aganglionic megacolon. HD is a developmental disorder of the intrinsic component of the enteric nervous system and it is characterized by the absence of ganglion cells in the myenteric and submucosal plexus [1]. These ganglion cells are responsible for normal peristalsis. Patients with HD usually present with functional intestinal obstruction at the level of aganglionosis. Most cases of HD involve the rectum or rectosigmoid region, but in 5% to 10% cases, the entire colon or part of small intestine may be involved [1]. Occurrence of HD is approximately 1 in 5,000 live births [2]. Males are more affected than females at a ratio around 4:1 [3,4]. 

In 80% to 90% cases of HD clinical features present soon after birth. HD should always be suspected when a new-born fails to pass meconium within 24 to 48 hours. Nearly 80% of patients show defecation problems along with delayed physical development, significant flatulence and emesis. While other HD patients usually don’t show any problems until late childhood, when these patients present with chronic constipation, malnutrition and developmental delays. Occasionally patients may suffer from diarrhoeas, that is, acute enteritis with a 30% fatality rate [5,6]. 

Transmural bowel biopsies have been considered an important diagnostic tool. Suction rectal biopsy is recommended in most centers as an outdoor procedure but its cost of instrument is a limiting factor for use. On hematoxylin and eosin (H&E) staining, lack of ganglion cells in the submucosal or intramuscular nerve plexus of the intestinal wall and hypertrophic nerve fibers and trunks are diagnostic of HD [7]. On immunohistochemistry (IHC) of patients who do not have HD, intrinsic nerves of muscularis mucosae and lamina propria show calretinin positivity. While in HD, this staining pattern is lost [8]. 

## Materials and methods

This study was a retrospective observational study conducted in the Department of Pathology in collaboration with the Department of Pediatric Surgery, Uttar Pradesh University of Medical Sciences (UPUMS), Saifai, Etawah, UP for three years (July 2017 to June 2020). Our institute is a tertiary health care centre of northern India, primarily providing health care to the rural and semi-urban population of this region. Ethical clearance was obtained from the institutional ethical committee. Necessary informed consent was obtained from included patient’s parents.

**Figure 1:**
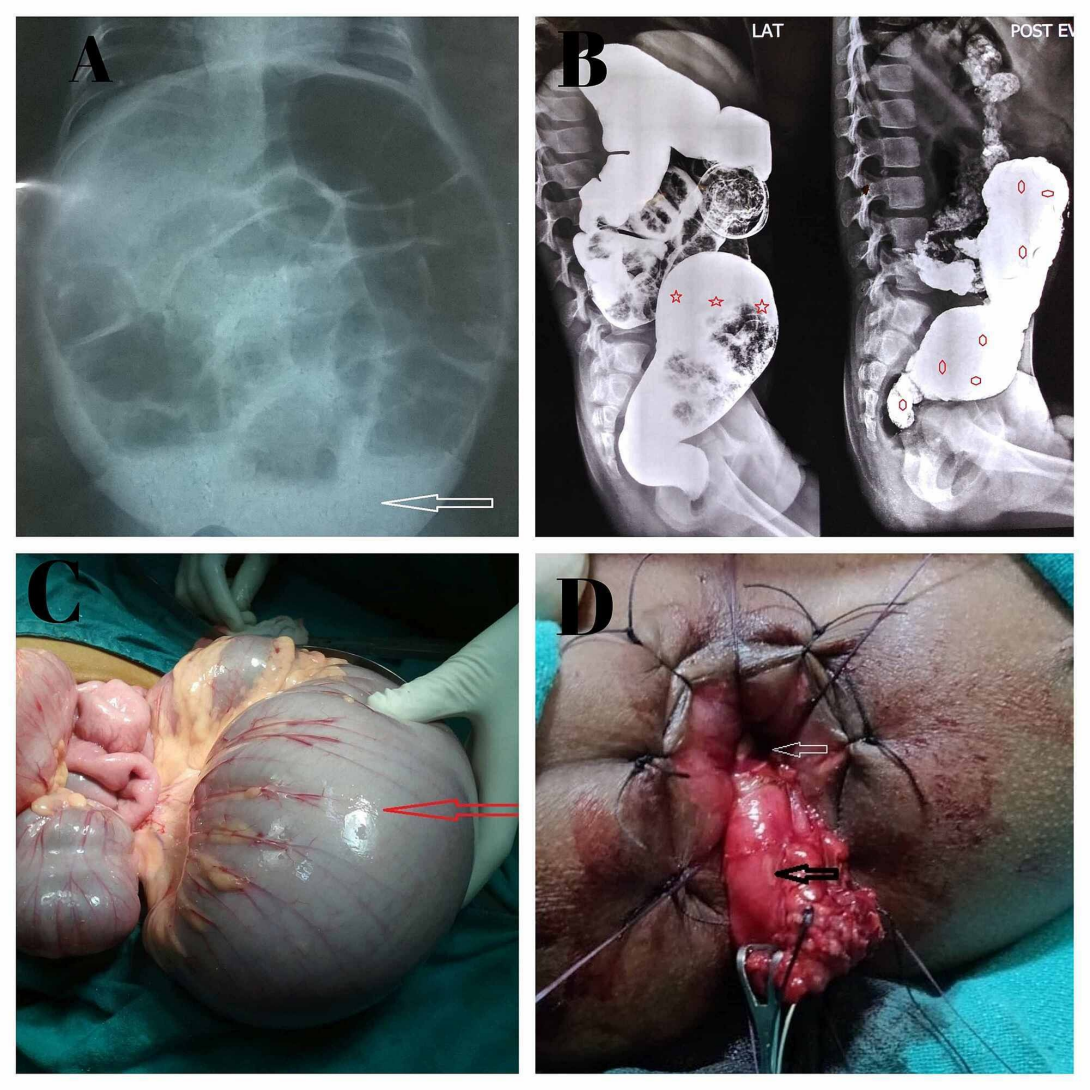
Image showing radiological and surgical features of HD. (A) Plain X-ray: paucity of air in the pelvic cavity (white arrow); (B) Contrast enema: sigmoid colon (star shape) is more dilated than rectum (reversal of rectosigmoid index) and 24-hour delayed film holding of dye in bowel (hexagonal shape); (C) intra-operative dilated sigmoid colon; (D) intra-operative retro rectal (white arrow retro rectal space) pull through (black arrow pull through bowel). HD: Hirschsprung’s disease.

Total 41 cases with the clinical presentation of neonatal intestinal obstruction to chronic progressive constipation in older children and whose radiological features (on X-ray and barium/contrast enema) were consistent with HD (e.g., paucity of air in pelvis, reversal of rectosigmoid index) were included in the study (Figure 1A, B). The intra-operative finding of dilated proximal bowel loop and spastic aganglionic segment were seen in all patients of this study (Figure 1C). All patients were initially managed with diverting leveling colostomy, that is, taking biopsy from the colostomy site. Multiple biopsies were taken from spastic segment, transition zone and proximal dilated segment.

**Figure 2:**
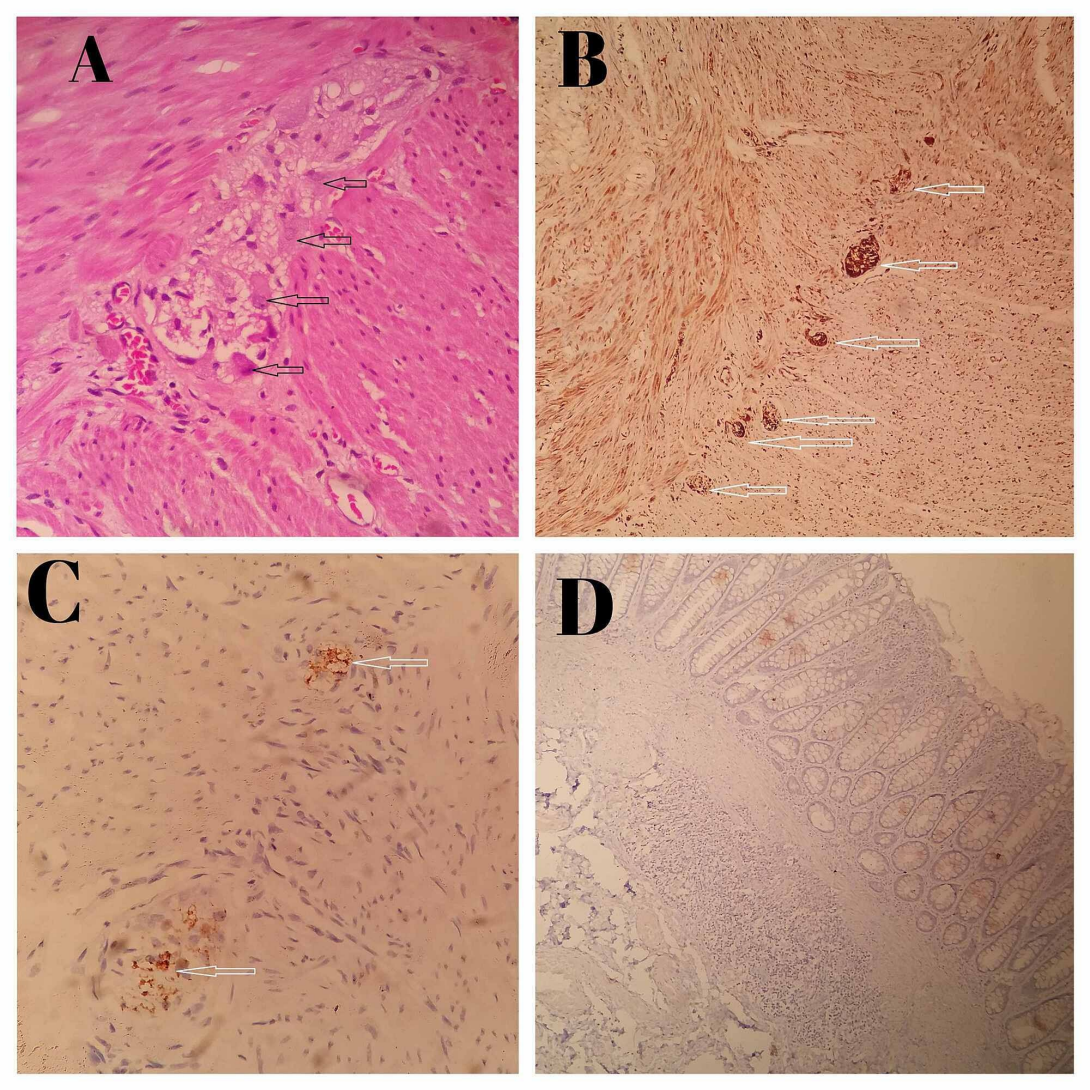
Microphotograph of histopathological examination and Immunohistochemistry finding of HD. (A) Micrograph 400X, H&E stain, ganglion cells (shown by black arrow) seen in NHD cases; (B) micrograph 100X, calretinin stain, ganglion cells (shown by white arrow) seen in NHD cases; (C) micrograph 400X, calretinin stain, ganglion cells (shown by white arrow) seen in suspected HD cases; (D) micrograph 100X, calretinin stain ganglion cells not seen in suspected HD cases. HD: Hirschsprung’s disease; NHD: non-Hirschsprung’s disease; H&E: hematoxylin and eosin.

The biopsy specimens were received in 10% formalin. After tissue processing, sections were submitted for microscopic examination. Multiple H&E-stained sections were examined and on the basis of presence or absence of ganglion cells, these were categorized into three diagnosis: HD, NHD and suspicion of HD (Table 1). Thereafter Calretinin immunostaining was applied (monoclonal rabbit IgG, Thermo Fisher Scientific, Cheshire, UK) on all the biopsy samples. These IHC slides were examined by two pathologists separately (Kappa value = 1). On examination of calretinin IHC slides, section from aganglionic segments (spastic) showed negative calretinin expression, while positive calretinin expression was seen in normal colon section (Figure 2A-D). After histopathology reports, patients with confirmed HD were managed by a definitive pull-through procedure (Figure 1D) while in NHD patient’s colostomy closure was done. 

**Table 1:** Histopathological and IHC criteria of classifying Hirschsprung’s disease. H&E: hematoxylin and eosin; IHC: immunohistochemistry; HD: Hirschsprung’s disease; NHD: non-Hirschsprung's disease.

Categories	Histologic features (H&E)	IHC features (calretinin)
HD	No ganglion cells (in spastic region), nerve hypertrophy	Absence of calretinin expression in nerve fibres
NHD	Ganglion cells present, no nerve hypertrophy	Staining present in submucosal nerve plexus, or in muscularis mucosa or in lamina propria
Suspicious HD	No ganglion cells, no nerve hypertrophy	No suspicion after evaluation of calretinin expression

Only cases with adequate biopsies samples were included in this study. Cases with inadequate biopsies, inadequate clinical details and autolysed specimen were excluded from the study.

## Results

During the study period of three years, total of 41 adequate biopsies were received from cases with clinical suspicion of HD. Out of these 41 cases, 30 cases were diagnosed as HD, on the basis of histopathological examination and calretinin IHC. The age of confirmed HD patients ranged from four days to 10 years (median age was six days). Twenty-two patients were in the neonatal period (Table 2). Constipation was the most common presenting symptom (96.7%) in confirmed HD cases in the study. Intestinal obstruction with or without constipation was the second most common symptom (80%) in HD patients. Only 23.3% of patients were presented with failure to thrive (Table 3). 

 

**Table 2:** Age group of confirmed Hirschsprung's disease (HD) patients.

Age of the patient (confirmed HD)	Number of patients	Percentage
0-28 days	22	73.33
1-12 months	05	16.67
>1 year	03	10.00
Total	30	

**Table 3:** Distribution of confirmed HD (n = 30) cases according to clinical presentation. *Failure to pass meconium or infrequent passage of stool. HD: Hirschsprung’s disease.

Clinical presentation	Number of cases	Percentage
Constipation*	29	96.7
Intestinal obstruction	24	80.0
Failure to thrive	07	23.3

On the basis of histopathology reports (H&E stained sections) out of the 41 cases, 25 (60.97%) cases were diagnosed as HD, 9 (21.95) cases as suspicious HD and 7 (17.07%) cases as negative for HD. IHC for calretinin was applied on all 41 cases (Table 4). After application of calretinin IHC, out of 41 cases, 30 (73.17%) cases were confirmed as HD while the remaining 11 (26.82%) cases were confirmed as NHD, hence all suspicious HD cases had been confirmed and categorized in HD and NHD groups (Table 4). Out of total 30 confirmed cases of HD, 24 (80%) patients were males while the remaining 6 (20%) cases were females and M/F ratio was 4:1. Twenty-two of the confirmed HD patients were neonates (<1 month age). Twenty-nine cases out of 30 confirmed HD cases were short segment HD. In our study sensitivity of calretinin positivity for ganglion cells was 100%, specificity was 68.75%, while positive and negative predictive values were 85.33% and 100%, respectively. Same findings were reconfirmed in tissue resected during the definitive procedure. 

**Table 4:** Hirschsprung’s disease diagnosis on basis of H&E staining and calretinin IHC. H&E: hematoxylin and eosin; IHC: immunohistochemistry; HD: Hirschsprung’s disease; NHD: non-Hirschsprung’s disease.

Categories	Based on H&E	Based on calretinin IHC
HD	25	30
NHD	7	11
Suspicious HD	9	0
Total	41	41

## Discussion

HD is one of the commonest causes of functional intestinal obstruction in children. Early diagnosis and timely surgical intervention are important to prevent disease associated with morbidity and mortality [9]. In our study, the majority of patients were males and male to female ratio (M/F) was 4:1. While many studies showed strong male predominance for example Ziad et al., found male/female ratio of 5.8/1 among HD patients [10]. Majority of HD patients (80%-90%) are diagnosed in the neonatal period [11]. In our study, 22 cases out of 30 (73.3%) were neonates. While in the study by Ziad et al. [10], they found 57% of their HD patients were neonates. In a study by Archibong, late presentation of patient was attributed to ignorance and poverty [9]. Clinical presentation of HD depends on the length of the aganglionic segment and severity of disease. Chief clinical presentations are delayed passage of meconium, intestinal obstruction, intestinal perforation, enterocolitis and constipation [12]. Intestinal obstruction is usually associated with distended abdomen, bilious vomiting, fever, dehydration, lethargy and not passing meconium. While in perforation, features of perforation peritonitis predominates, and in neonates with this condition, colon must be thoroughly examined and appropriate biopsies should have been taken [12]. In our study, among confirmed HD cases, most of the cases (96.7%) presented with constipation (failure to pass meconium in neonates and infrequent passage of stools in older patients) and second most common (80%) clinical presentation was intestinal obstruction. 

About 70% to 75% of HD can be diagnosed with barium or contrast enemas. However classical radiological features are absent in about 25% of neonates [13]. Biopsy is the gold standard for the diagnosis of HD [12]. Suction rectal biopsy (SRB) is an important tool available for outpatient procedure. In our study, we performed full-thickness biopsy, because patients came in sick condition and urgent laparotomy was necessary. Required instruments essential for SRB and facility for study of acetylcholinesterase (AChE) were not available in our institute. We explored all the patients on the basis of clinical history and radiological feature of this study and took multiple full-thickness biopsies. Surgery had been performed in all the patients in our study. Colostomy with multiple biopsies was done as primary procedure. After confirmation of diagnosis by histopathology and IHC, definitive pull-through and/or colostomy closure was done. Studies showed that Duhamel’s endorectal pull-through should be the preferred surgical treatment with least complications [14,15].

In histopathological examination, lack of ganglion cells in submucosal nerve plexus is required for diagnosis of HD. In our study after evaluation of H&E-stained slides, we confirmed 25 cases as HD and seven cases as NHD, while nine cases termed as suspected HD. We used calretinin immunostaining in all cases. In our study all NHD cases expressed calretinin positivity, while all HD cases (30 cases) showed negative for calretinin. In a study, Zuikova et al. [16] stated that calretinin IHC is easier to interpret than AChE. Barshack et al. [8] also concluded that aganglionic segments (spastic) shows negative calretinin expression, while positive calretinin expression is seen in normal colon section. Maldyk et al. [17] also confirmed in their study that calretinin expression was positive in all rectal biopsies with ganglionic cells, while expression was negative in all aganglionic segments. In our study sensitivity of calretinin positivity for ganglion cells was 100%, specificity was 68.75%, while positive and negative predictive values were 85.33% and 100%, respectively. Mukhopadhyay et al. [18] stated that sensitivity of calretinin positivity for ganglion cells was 100%, specificity was 97.44%, while positive and negative predictive values were 84.62% and 100%, respectively.

In a study by Gonzalo and Plesec [19], all patients without HD had calretinin positive nerve fibres in lamina propria or muscularis mucosa and all patients with HD showed no calretinin staining of nerve fibres in the same regions. Guinard-Samuel et al. [20] also noticed 12 cases as suspicious for HD And these suspicious cases were accurately diagnosed by calretinin immunohistochemistry. In the present study, after evaluating calretinin IHC on suspicious cases for HD, we confirmed HD in five of these suspicious cases, because of negative calretinin expression. Rest of the four cases showed calretinin positivity, hence confirmed as NHD. 

The limitation of our study is a relatively small sample size. In the study duration of three years, we diagnosed only 30 confirmed HD cases. Further studies with larger sample are desirable. 

## Conclusions

Our study showed male predominance in confirmed HD cases. Constipation and intestinal obstruction were the most common clinical presentation in these cases. The study also concludes that calretinin immunostaining is very useful to diagnose suspected HD cases. Calretinin IHC should be routinely used in all suspicious cases of HD.
